# Greenlight laser (XPS^TM^) 180W prostatectomy for treatment of benign prostate hyperplasia in patients with uncorrectable bleeding tendency

**DOI:** 10.1080/2090598X.2022.2156655

**Published:** 2022-12-15

**Authors:** Ahmed M. Elshal, Fady K. Ghobrial, Mahmoud Laymon, Mohamed Elegeezy, Ahmed R. El-Nahas

**Affiliations:** a Urology Department, Urology and Nephrology Center, Mansoura University, Egypt; b Department of Urology, Faculty of Medicine, Damietta University, Egypt; c Department of Internal Medicine, Hepatology Unit, Mansoura University, Al Mansurah, Egypt

**Keywords:** Prostate, Greenlight, vaprization, hepatic, bleeding tendency

## Abstract

**Objectives:**

Safety of GreenLight™ laser prostatectomy (GL-LP) in patients with ongoing blood thinners has been proven. Yet, the possibility of drug manipulation makes it a less challenging situation compared to treating patients with uncorrectable bleeding tendency. Herein, we aim at evaluating the outcomes of XPS™-180 W GL-LP for treatment of BPH in patients who had uncorrectable bleeding tendency due to hepatic dysfunction.

**Methods:**

A prospectively maintained database for all patients who underwent GL-LP for symptomatic BPH was reviewed. Patients were divided into two groups based on the degree of hepatic dysfunction using Fib-4 index: Group 1 (indexed patients; low-risk Fib-4) and Group 2 (non-indexed patients; intermediate-high-risk Fib-4) included those who had chronic liver disease associated with either thrombocytopenia and/or hypoprothrombinemia. Primary outcome was the difference in perioperative bleeding complications between the two groups. Other outcome measures included all perioperative findings and complications as well-functional outcome measures.

**Results:**

The study included 140 patients (93 indexed patients and 47 non-indexed). There were no significant differences between both groups in operative time, laser time and energy, auxiliary procedures, catheter time, hospital stay, and hemoglobin deficit. The need for blood transfusion was significantly more in group 2 (two patients (4.3%) versus no patients in group 1, P = 0.045). Perioperative and late postoperative complications were comparable for both groups (P = 0.634 and 0.858, respectively). There were no significant differences in the postoperative uroflow, symptoms score, and PSA reduction between the two groups (P = 0.57, 0.87, and 0.05, respectively).

**Conclusions:**

XPS™-180 W GL-LP is a safe and effective technique for treatment of BPH in patients with uncorrectable bleeding tendency due to hepatic dysfunction.

## Introduction

Surgical treatment of lower urinary tract symptoms (LUTS) caused by benign prostatic hyperplasia (BPH) of less than 80 gm has been changed from monopolar transurethral resection of the prostate (TURP) to transurethral resection in saline (TURIS) or vaporization techniques using Plasma kinetic or Laser energies [1]. GreenLight Photoselective vaporization prostatectomy (GL.PVP) was proved to be safe and effective treatment option [2]. As per Goliath trial, GreenLight laser PVP is non-inferior to TURP in an index case [3].

The high prevalence of chronic liver disease (CLD) in elderly Egyptians is attributed to past endemics of bilharziasis and hepatitis C virus infections leading to liver fibrosis and cirrhosis [4,5]. Patients with CLD represent a challenge to all surgical interventions because of the increased risk of intra and postoperative complications [6]. In surgical treatment of BPH, there is a concern about bleeding secondary to platelet deficiency, malfunction or both, and decreased prothrombin activity among these patients [7]. The advantages of GreenLight™ laser prostatectomy (GL-LP) might be more obvious in non-index patients, especially those with increased risk of bleeding. GL-LP has the advantage of being safer for patients using antiplatelets or anticoagulants [8–13]. To the best of our knowledge, the safety of XPS/GL-LP in patients with bleeding tendency secondary to CLD was not previously reported.

This study was conducted to evaluate the outcomes of XPS-GreenLight™laser 180 W prostatectomy for the treatment of BPH in patients who had bleeding tendency due to hepatic dysfunction.

## Patients and methods

### Study design

A retrospective analysis of a prospectively maintained database of all consecutive patients who underwent GL-LP for symptomatic BPH between August 2017 and March 2019 was conducted. Patients with refractory LUTS secondary BPO or patients with urine retention secondary to BPO who failed medical therapy were eligible for inclusion in this study. They were enrolled in the study after obtaining written informed consent. Patients were not included if they had a neurological disorder, ongoing anticoagulants, or antiplatelet medications. Patients with a history of previous prostate surgery and patients diagnosed with prostate cancer were excluded. This study was performed in line with the principles of the Declaration of Helsinki.

The degree of hepatic fibrosis in patients with chronic liver disease was calculated using Fib-4 index (age [yr] × AST [U/L])/((PLT [10^9^/L]) × (ALT [U/L])^1/2^) [14]. They were categorized into low-, intermediate-, and high-risk groups for advanced fibrosis based on the following suggested cut-offs: <1.30 (low risk), 1.30–2.67 (intermediate risk) and >2.67 (high risk) [15]. Patients were divided into two groups based on the degree of hepatic dysfunction using Fib-4 index: Group 1 (indexed patients; low-risk Fib-4) and Group 2 (non-indexed patients; intermediate-high-risk Fib-4).

### Study workup

Preoperative workup included international prostate symptom score (IPSS), clinical examination, uroflowmetry, and post-voiding residual (PVR) estimation. Laboratory tests included complete blood count (CBC), hepatitis viral serology, prothrombin concentration, and prostate-specific antigen (PSA) estimation. Radiological investigations included trans-abdominal ultrasound and transrectal ultrasound (TRUS) ± prostate biopsy (as indicated by PSA and digital rectal exam). Baseline assessment includes IPSS, Qol score, Qmax, and PVR and follow-up was scheduled at 1, 3, 6, and 12 months and then annually.

Patients were divided into two groups according to bleeding tendency due to hepatic dysfunction. Group 1 (indexed patients) were those with normal coagulation profile. Group 2 (non-indexed patients) included those who had chronic liver disease (CLD) associated with either thrombocytopenia (platelet count <150,000/µl) [16], reduced prothrombin concentration (<70%), or both.

### Intervention

Procedures were performed with patients under spinal anesthesia, and preoperative antibiotic prophylaxis was administered according to international guidelines. Urethrocystoscopy was performed for morphological assessment. XPS-180 W GreenLight™ Laser machine (AMS, Minnetonka, MN, USA) with MoXy™ fibers was used for all patients. All procedures were performed by two expert surgeons (AME and ML) who had passed the learning curve of GL-LP. According to prostate size and morphology as well as surgeon preference, the procedure was performed using technique of GL.PVP or GL.PVEP. In both techniques, the procedure started by creation of a working channel from the bladder neck till the verumontanum using 80 W power. In GL.PVP, prostatic lobes were vaporized as previously described till reaching the surgical capsule with gradually increasing the laser power to 180 W [17]. In GL.PVEP, vaporization of partially enucleated prostatic adenoma was performed as previously described [18,19].

### Outcomes measures

Primary outcomes were differences in bleeding complications (intraoperative and postoperative), and hemoglobin and hematocrit deficit between the two groups. Secondary outcomes were time to catheter removal, length of hospital stay, and postoperative complications. All complications were classified based on a panel of GreenLaser users according to a modified Clavien classification [20]. The efficacy of the treatment was evaluated with IPSS, Qol score, Qmax, PVR, incidence of postoperative retention requiring catheterization, and the need for re-intervention. Postoperative PSA reduction was counted (preoperative PSA minus nadir postoperative PSA/preoperative PSA × 100) and compared.

### Statistical analysis

Data were collected and analyzed using SPSS v21 statistical program. Continuous data were expressed as mean and standard deviation (SD) and compared between both groups using independent sample t-test or expressed as median and range and compared with Mann Whitney U-test as appropriate. Nominal data were expressed as numbers and percentages and compared using chi-square or Fisher’s exact test as appropriate. P value <0.05 was considered statistically significant.

## Results

The study included 140 patients with a median prostate size of 65 (30–125 ml). Patients were 93 indexed patients, in group 1, and 47 were non-indexed, in group 2. Table 1 summarizes preoperative patients and prostatic characteristics. Table 2 summarizes operative and early postoperative outcomes. There were no significant differences between both groups in operative time, laser time, energy used and auxiliary procedures. Similarly, mean catheter time, hospital stay, and hemoglobin deficit were comparable between both groups.

**Table 1.** Preoperative patients’ and prostatic characters of the studied groups.

	Group 1 Indexed 93 patients	Group 2 Non-indexed 47 patients	P Value
Mean age at time of surgery (SD)	64.8 (7.4)	64.8 (7.6)	0.995
Number of patients with ASA Score ≥III (%)	15 (16)	19 (40.4)	0.002
Mean IPSS in non-catheter-dependent patients (SD)	23.4 (5.3)	25.3 (5.3)	0.278
Number of patients with hypoprothrombinemia (%)	0	25 (53.2)	<0.0001
Number of patients with thrombocytopenia (%)	0	35 (70.5)	<0.0001
Mean preoperative platelets count ×10^3^ (SD)	225 (66)	121 (48)	<0.0001
Number of patients with anti-HCV antibodies (%)	26 (28)	32 (68)	<0.0001
Number of patients with liver cirrhosis by TAUS (%)	9 (9.7)	24 (51)	<0.0001
Number of patients with splenomegaly by TAUS (%)	5 (5.4)	22 (46.8)	<0.0001
Mean TRUS-estimated prostate volume (SD)	66.9 (19.8)	65.2 (21)	0.631
Indication for surgery Indwelling urethral catheter LUTS Recurrent hematuria	34 (36.4) 56 (60.2) 3 (3.2)	13 (27.7) 31 (66) 3 (6.4)	0.443

ASA score; American Society of Anesthesiologists score, Anti-HCV; hepatitis-C virus antibodies, TAUS; transabdominal ultrasound, TRUS; Transrectal ultrasound, HB; hemoglobin, HCV; hematocrit value

**Table 2.** Operative data and early postoperative outcomes.

	Group 1Indexed 93 Patients	Group 2Non-indexed47 Patients	P Value
**Operative Data**			
Mean operative time in minutes (SD)	78 (29.5)	74.3 (34.2)	0.509
Mean lasing time in minutes (SD)	45.5 (17.3)	45.9 (20.7)	0.899
Mean laser energy in KJ (SD)	382.3 (145.8)	390.8 (168.5)	0.765
GreenLight laser procedure: N (%) PVP PVEP	76 (81.7) 17 (18.3)	35 (74.5) 12 (25.5)	0.317
Laser lithotripsy of vesical stones: N (%)	13 (14)	6 (12.8)	0.843
Auxiliary procedures: N (%) Transurethral resection of residual prostatic tissue Vapo-enucleation and morcellation of median lobe	1 (1.1) 2 (2.2)	1 (2.1) 0 (0)	0.533
**Early Postoperative Outcomes**			
Mean time to catheter removal in days (SD)	1.3 (0.6)	1.3 (0.5)	0.581
Median HB-Deficit (range)	0.6 (−0.8:3)	0.6 (−0.1:5)	0.722
Median HCV-Deficit (range)	1.7 (−2.9:9.7)	2.3 (−3:15.6)	0.459
Mean hospital stay in days (SD)	1.3 (0.6)	1.3 (0.7)	0.527

Table 3 presents intraoperative, early postoperative (perioperative), and late postoperative complications according to modified Clavien classifications and management of these complications. The need for blood transfusion was significantly higher in group 2, and it was needed in two patients (4.3%) versus no patients in group 1 (P = 0.045). The incidence of failed first trial of voiding after urethral catheter removal and the incidence of acute urine retention (AUR) during the first postoperative 30-days were comparable for both groups (Table 3).

**Table 3.** Perioperative and delayed postoperative complications.

Complication	Modified Clavien grade	Group 1 Indexed 93 Patients No (%)	Group 2 Nonindexed 47 Patients No (%)	P Value	Management
Perioperative (Intraoperative and 1^st^ 30 days)*		**13 (14)**	**8 (17)**	**0.634**	
Fever (UTI)	I	2 (2.2)	0 (0)		Cold fomentations & antipyretics
Capsular violation	II	3 (3.2)	2 (4.3)		Prolonged catheter drainage
Postoperative hematuria	II	3 (3.2)	1 (2.1)		Conservative (Prolonged CBI)
Failed 1^st^ TOV	II	1 (1.1)	1 (2.1)		Catheterization then repeat voiding trial
AUR during 1^st^ month	II	2 (2.2)	2 (4.3)		Catheterization then repeat voiding trial
Epididymo-orchitis	II	1 (1.1)	1 (2.1)		Antibiotics
Intraoperative bleeding	IIIa	3 (3.2)	2 (4.3)		Coagulation using TUR loop
Myocardial infarction	IVa	1 (1.1)	1 (2.1)		ICU measures
**Delayed postoperative (after 30 days)**		**9 (9.5)**	**5 (10.6)**	**0.858**	
Persistent LUTS	II	2 (2.2)	1 (2.1)		Alpha blockers
LUTS with residual prostate adenoma	IIIa	4 (4.3)	2 (4.3)		TURP for the adenoma
Bladder neck contracture	IIIa	2 (2.1)	1 (2.1)		Bladder neck incision
Urethral stricture	IIIa	1 (1.1)	0		Visual internal urethrotomy
Persistent stress urine incontinence	IIIa	0	1 (2.1)		Mid-urethral sling

*Some patients had more than one complication, UTI; Urinary tract infection, TOV; trial of voiding, AUR; acute urine retention, BOO; bladder outlet obstruction, LUTs: lower urinary tract symptoms, CBI continuous bladder irrigation.

After a median follow-up of 13.3 months (range 6.1–65.7), the mean Qmax was 21 ± 9 and 20 ± 9 ml/second in indexed and non-indexed patients, respectively (P = 0.572). The median IPSS, QoL, and PVR were 4, 1, and 15 ml for indexed patients and 5, 1, and 13.5 ml for non-indexed patients (P = 0.879, 0.880, and 0.830 respectively). The need for surgical reinterventions in the form of TURP for residual prostatic adenomas, incision for bladder neck contracture, urethrotomy for urethral stricture, and urethral sling were comparable for both groups (7.5% vs 8.5%). The mean percent reduction of PSA (SD) during follow-up was 66 (23)% in indexed patients vs 53 (25)% for non-indexed patients (P = 0.057).

## Discussion

The present study has proven the safety and efficacy of XPS/GL-LP for surgical treatment of BPH in patients with bleeding tendency secondary to CLD. Intraoperative bleeding requiring coagulation using TURP loop was observed in 3.2% and 4.3% in indexed and non-indexed patients. While postoperative hematuria was encountered in 3.2% and 2.1%, respectively. These differences were not statistically significant. However, blood transfusion was needed in the two patients in non-indexed patients versus none of the indexed patients (P = 0.045). The reported hemorrhagic complications during XPS-GreenLight™ 180 W Laser prostatectomy in patients with ongoing blood thinners were variably reported [9].

Table 4 summarizes outcome of GL.XPS in patients with ongoing variable blood thinners [9,11,21–24]. On the contrary, Sachs et al. reported no hemorrhagic complications after PVP for 20 patients under novel anticoagulants using different GL.PVP generations in 3 centers [10].

**Table 4.** Outcome of contemporary series of GL.XPS in patients with ongoing blood thinners.

Study	Ongoing blood thinners state	Number (%)	Prostate size (ml)	Hgic. AEs No. (%)	Blood Tx No. (%)	Hospital stay(days)	Catheter time (days)	HgB deficit (gm/dl)	Overall AEs No. (%)
Meskawi et al. 2019	Control	274	76 (67)	24 (8.8)	0	0.4	1 d	0.9	85 (31)
	ASA	87	71 (61)	9 (10.3)	1 (1.1)	0.5	1.2	0.9	25 (28.7)
	Non-ASA antiplatelet	24	87 (86)	6 (25)	1 (4.2)	2.9	1.7	0.9	11 (45.8)
	anticoagulants	37	72 (61)	11 (29.7)	0 (0)	1.3	1.4	0.8	17 (45.9)
Eken et al. 2018	Control	174	57.2 (27:185)	7 (4.0)	0	NR	NR	NR	49 (28.2)
	Ongoing ASA	59							
	Non-ASA antiplatelet→ LMWH shift	9		3 (5.1)					19 (32.2)
	anticoagulants→ LMWH shift	11							
Hueber et al. 2015	Non-ASA antiplatelet	53/1196 (4.4)	61.4	60/618 (9.7)	0	1	1	NR	No effect for blood thinners
	anticoagulants	76/1196 (6.4)							
Lee et al. 2016	Control	198	54.1 (40–109)	8 (3.8)	0	3.4 (1–4)	2 (1–2)	NR	23 (11.6%)
	ASA	146	70.5 (44–100)	9 (4.9)	0	4 (1–4)	2 (1–2)	NR	25 (13.4%)
	Non-ASA antiplatelet	34							
	anticoagulants	57							
Knapp et al. 2017	Control	272	79.1(47)	5 (1.8)	0	0.95 (0.66)	0.65 (0.8)	NR	54 (19.9)
	Ongoing ASA	59	90.8 (58.7)	1 (1.7)	0	1.9 (3.3)	1.3 (3.1)	NR	10 (23.8)
	Non-ASA antiplatelet								18 (30.5)
	anticoagulants								
Waters et al. 2021	Non-ASA antiplatelet and or anticoagulants	374	NR	10 (6.3)	2 (0.5)	2.7 (1–14)	NR	NR	24 (6.42)

Hgic; hemorrhagic, AEs.; adverse events, HgB; hemoglobin, ASA; acetylsalicylic acid, LMWH; low molecular weight heparin NR; not reported

The other primary endpoint in the present study was hemoglobin deficit. There was no significant difference between indexed and non-indexed patients (P = 0.772). The same was reported by Sohn et al. when they compared patients who stopped anticoagulant medications before GL.PVP with those who continued them [13] and spernatet al who evaluated GL.PVP in patients receiving clopidogrel [8]. The safety of GL.PVP in patients with bleeding tendency because of either CLD or continuing antiplatelets or anticoagulants is attributed to its absorption by oxyhemoglobin leading to an effective coagulated zone adjacent to the vaporization one [12].

Perioperative complications frequency in indexed and non-indexed patients in the present study were comparable (14% vs 17%, P = 0.634). Moreover, the severity of these complications was also comparable as grades 3 and 4 were observed in 4.3% and 6.4% for indexed and non-indexed patients, respectively (P = 0.594). Many studies reported comparable incidence of perioperative complications between patients receiving and not receiving anticoagulants [9,11] or antiplatelets [8].

The efficiency of successfully completing XPS-GreenLight™ 180 W Laser prostatectomy in non-indexed patients was proved by lack of significant statistical differences between both groups in laser time, operative time, catheter time, and hospital stay (Table 2). Similarly, Knapp et al. had found comparable results for laser time and operative time, but significantly longer catheter period and hospital stay in anticoagulated patients when compared with controls [11]. The short-term success rates were comparable between both groups for the failed first trial of voiding, need for re-catheterization to manage AUR (Table 3).

The long-term outcomes as evaluated with IPSS, QoL score, Qmax, PVR, and percent reduction of PSA were also comparable in both groups. These results were in consistency with long-term functional outcomes reported by Ajib et al. for XPS-GreenLight™ 180 W Laser prostatectomy [25]. The reintervention rates during follow-up for residual adenoma and bladder neck contracture were similar between both groups (4.3% and 2.1%, respectively), while reintervention for urethral stricture and urinary incontinence was needed in one patient of each group. The overall need for reintervention were 7.5% and 8.5% for indexed and non-indexed patients, respectively. A lower reoperation rate of 4.8% was reported by Calves et al. after a long-term follow-up for XPS-GreenLight™ 180 W Laser prostatectomy [26]. However, calves et all relied on patients` reported outcomes (subjective) via a non-validated questionnaire.

Limitations of this study include the retrospective analysis that may have led to selection bias of doing non-indexed patients by more experienced surgeons. The strong points of this study are the prospective maintenance of the database, long-term follow-up, and classification of complications according to the system developed specifically for evaluating GreenLight Laser prostatectomy.

XPS-GreenLight™ 180 W Laser prostatectomy is a safe and effective technique for treatment of patients with bleeding tendency due to hepatic dysfunction.

## Abbreviation list

AUR: acute urine retention; BPH: benign prostatic hyperplasia; CLD: chronic liver disease; GL.PVEP: Greenlight XPS photoselective vapo-enucleation of the prostate; GL.PVP: greenlight laser XPS photoselective vaporization of the prostate; GL/XPS: greenlight laser extreme power system; GL-LP: greenlight laser prostatectomy; IPSS: international prostate symptoms score; LUTs: Lower urinary tract symptoms; OP: open prostatectomy; PSA: prostate-specific antigen; PVR: post-void residual; Q.max: maximum uroflow; QoL: quality of life; TOV: trial of voiding; TRUS: transrectal ultrasound; TURIS: transurethral resection of the prostate in saline; TURP: transurethral resection of the prostate
